# Adénocarcinome de l'ouraque: une cause rare d'hématurie

**DOI:** 10.11604/pamj.2013.14.8.1050

**Published:** 2013-01-06

**Authors:** Issam En-nafaa, Rachida Latib, Taoufik Africha, Ilham Chami, Najib Boujida, Leila Jroundi

**Affiliations:** 1 Service de radiologie, INO. CHU Rabat-Salé, Maroc

**Keywords:** Hématurie, ouraque, adénocarcinome, Hematuria, urachus, adenocarcinoma

## Abstract

Les cancers de l'ouraque sont rares et de pronostic sombre. Les auteurs rapportent un cas d'adénocarcinome de l'ouraque révélé par une hématurie et exploré par échographie et tomodensitométrie abdominopelvienne.

## Introduction

Les tumeurs de l'ouraque sont rares, elles représentent 0,01% des cancers de l'adulte et 0,17 à 0,34% des cancers de vessie. Ce sont des tumeurs très agressives avec un pronostic très sombre. Nous rapportons un nouveau cas d'adénocarcinome de l'ouraque chez un adulte jeune.

## Patient et observation

Un jeune homme de 30 ans, sans antécédent pathologique notable, a consultait pour une hématurie terminale associée à des troubles urinaires à type de pollakiurie et impériosité mictionnelle sans fièvre. L'examen clinique était sans particularité.

**Figure 1:**
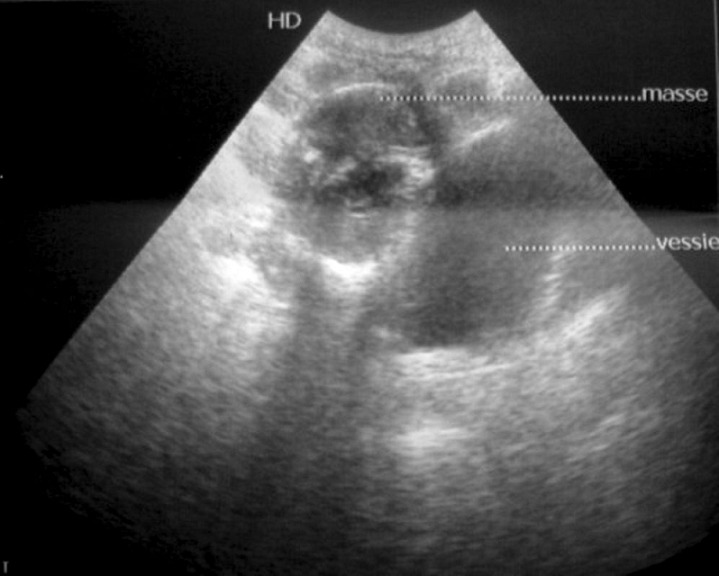
Echographie abdominopelvienne en coupes sagittales centrées sur la vessie

Une échographie abdominale a été réalisée et a montré la présence d'une masse médiane, du dôme vésicale, tissulaire, hypoéchogène hétérogène, renfermant des calcifications (Figure 1).

**Figure 2:**
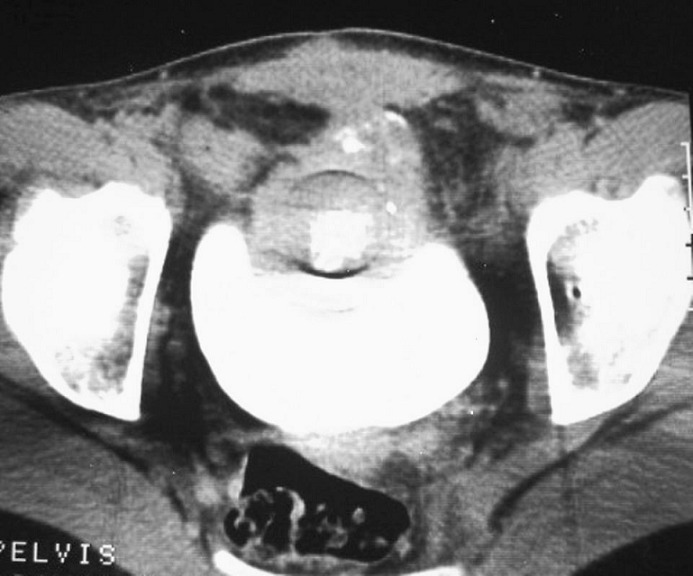
TDM abdomino-pelvienne en coupes axiales de 5 mm d′épaisseur, fenêtre parenchymateuse, après injection de produit de contraste

Une tomodensitométrie abdominopelvienne en coupes axiales de 5 mm d’épaisseur avant et après injection de produit de contraste a objectivé la présence d'un processus tumoral se développant à partir de la paroi antérieure de la vessie, de densité tissulaire, contenant des calcifications aussi bien périphériques que centrales et se rehaussant de façon hétérogène après injection de produit de contraste. Ce processus mesure 70 X 55 mm, se prolabant dans la vessie avec un développement exoluminal (Figure 2).

Le diagnostic d'une tumeur de l'ouraque a été posé. Le patient a été opéré avec une chimiothérapie postopératoire et l'examen anatomo-pathologique a confirmé qu'il s'agit d'un adénocarcinome de l'ouraque.

## Discussion

L'ouraque est décrit comme un tube reliant le dôme vésical à l'ombilic se formant à partir du 28e jour gestationnel, il repose entre le péritoine et le fascia transversalis, en dedans de l'espace de Retzius et s’étend du dôme vésical à l'ombilic. Sa longueur varie de 3 à 10 cm et son diamètre de 8 à 10 mm. Il est accompagné des ligaments ombilicaux, résidus des artères ombilicales [1].

La première description d'une tumeur de l'ouraque a été faite en 1863 par Hue et Jacquin. Le cancer de l'ouraque est très rare, il représente 0,01% des cancers de l'adulte et 0,17 à 0,34% des cancers de vessie. Elle peut survenir à tout âge, avec des extrêmes de 6 à 85 ans et semble toucher le plus souvent le sexe masculin (65 à 80% des cas), âgé de 40 à 70 ans dans 68% des cas [1].

Mostofi et al [2] ont précisé des critères diagnostiques pour l'adénocarcinome de l'ouraque: Tumeur localisée au niveau du dôme ou la paroi antérieure de la vessie; Envahissement de la vessie de dehors en dedans avec muqueuse vésicale intacte ou ulcérée; Absence de cystite kystique ou glandulaire; Présence de reliquat embryonnaire.

La pathogénie n'est pas claire, il s'agit très probablement d'une métaplasie de l’épithélium transitionnel due à une infection chronique [3]. La symptomatologie clinique est dominée par l'hématurie qui se voit dans 65 à 85% des cas. On peut avoir une masse sus-pubienne sensible, des douleurs abdominales, des troubles mictionnels, un suintement ombilical, des sécrétions urinaires muqueuses (25% des cas), hautement évocatrices d'un adénocarcinome mucosécrétant mais non spécifiques de l'ouraque, et une déformation ombilicale [1].

Le diagnostic différentiel se pose avec l'adénocarcinome primitif de la vessie ou métastase vésicale d'un cancer prostatique, ovarien ou colique [4]. L'urographie intraveineuse est généralement normale surtout à un stade précoce et permet de rechercher une déviation urétérale, un effet de masse supra vésical ou des calcifications pelviennes [1].

L’échographie vésicale précise les critères échographiques de la masse médiane, permet de détecter un envahissement vésical et de rechercher d’éventuelles localisations métastatiques. La cystoscopie couplée à une palpation abdominale met en évidence, dans 90% des cas, une tumeur du dôme ou de la face antérieure de la vessie avec précision de sa taille et sa mobilité et permet de faire une biopsie transurétrale [1].

La TDM est déterminante pour apporter le diagnostic positif de la tumeur, préciser sa nature solide, kystique ou mixte, la présence de calcifications qui sont généralement périphériques mais peuvent être également centrales ou les deux comme c'est le cas chez notre patient. Elle précise l'envahissement vésical (présent dans la majorité des cas), locorégional ganglionnaire ou métastatique. Elle permet d'effectuer des biopsies guidées et de détecter d’éventuelles récidives après traitement [1, 5].

L'imagerie par résonance magnétique (IRM) n'apporte pas de renseignements morphologiques supplémentaires par rapport au scanner. Elle peut être utile en cas de contre-indication au produit de contraste ou s'il existe un doute diagnostique [5].

Du fait de l’évolution insidieuse de la tumeur, le diagnostic se pose en général tardivement ce qui influence le pronostic. Dans une série de 25 cas Thali-Schwab et al [5] rapporte 28% des patients avait des métastases au moment du diagnostic de la tumeur et c'est également le cas de notre patient où des métastases pulmonaires ont été découvertes.

Les sites fréquents de métastases sont représentés par les ganglions régionaux, le foie, le poumon et l'os. D'autres localisations sont également possibles comme le cerveau, la cavité péritonéale, les ovaires et le tissu mou périrectal.

Le traitement consiste à une exérèse partielle ou totale de la vessie incluant l'ouraque et l'ombilic, associée à une lymphadénectomie pelvienne, avec possibilité de voie laparoscopique [1, 5]. L'association avec la chimiothérapie et la radiothérapie est intéressante dans les stades métastatiques [1].

Le pronostic de l'adénocarcinome de l'ouraque est péjoratif avec 25 à 45% de survie à 5ans [4]. La récidive locale ou métastatique est fréquente. Le CA 125 serait un marqueur utile pour le suivi postopératoire, un taux élevé est un argument en faveur d'une chimiothérapie complémentaire [1].

## Conclusion

L'adénocarcinome de l'ouraque, bien que rare, doit être inclus dans le diagnostic différentiel d'hématurie, permettant ainsi le diagnostic positif et une prise en charge adéquate.
